# Summer-Long Grazing of High vs. Low Endophyte (*Neotyphodium coenophialum*)-Infected Tall Fescue by Growing Beef Steers Results in Distinct Temporal Blood Analyte Response Patterns, with Poor Correlation to Serum Prolactin Levels

**DOI:** 10.3389/fvets.2015.00077

**Published:** 2015-12-21

**Authors:** Joshua J. Jackson, Merlin D. Lindemann, James A. Boling, James C. Matthews

**Affiliations:** ^1^Department of Animal and Food Sciences, University of Kentucky, Lexington, KY, USA

**Keywords:** *Neotyphodium coenophialum*, ergot alkaloids, blood metabolites, blood cells, cattle

## Abstract

Previously, we reported the effects of fescue toxicosis on developing Angus-cross steer growth, carcass, hepatic mRNA, and protein expression profiles of selected serum proteins, and blood clinical and chemical profiles, after summer-long grazing (85 days) of high endophyte (HE)- vs. low endophyte (LE)-infected fescue pastures. We now report the temporal development of acute, intermediate, and chronic responses of biochemical and clinical blood analytes determined at specified time intervals (period 1, day 0–36; period 2, day 37–58; and period 3, day 59–85). Throughout the trial, the alkaloid concentrations of the HE forage was consistently 19–25 times greater (*P* ≤ 0.002) than the concentration in the LE forage, and HE vs. LE steers had continuously lower (*P* ≤ 0.049) serum prolactin (85%), cholesterol (27%), and albumin (5%), but greater red blood cells (7%). The HE steers had decreased (*P* = 0.003) ADG only during period 1 (−0.05 vs. 0.4 kg/day). For period 1, HE steers had reduced (*P* ≤ 0.090) numbers of eosinophils (55%) and lymphocytes (18%), serum triglyceride (27%), and an albumin/globulin ratio (9%), but an increased bilirubin concentration (20%). During period 2, serum LDH activities were 18% lower (*P* = 0.022) for HE vs. LE steers. During period 3, serum levels of ALP (32%), ALT (16%), AST (15%), creatine kinase (35%), glucose (10%), and LDH (23%) were lower (*P* ≤ 0.040) for HE steers. Correlation analysis of serum prolactin and other blood analytes revealed that triglycerides (*P* = 0.042) and creatinine (*P* = 0.021) were moderately correlated (*r* ≤ 0.433) with HE serum prolactin. In conclusion, three HE-induced blood analyte response patterns were identified: continually altered, initially altered, and subsequently “recovered,” or altered only after long-term exposure. Blood analytes affected by length of grazing HE vs. LE forages were either not or poorly correlated with serum prolactin. These data reveal important, temporal, data about how young cattle respond to the challenge of consuming HE pasture.

## Introduction

Tall fescue (*Lolium arundinaceum*) is a forage grass that is commonly used by many livestock producers in the southeastern United States. The hardiness of the grass is primarily due to the fact that the majority of the fescue in the southeast is infected with the endophytic fungus, *Neotyphodium coenophialum*. Unfortunately, cattle consuming endophytic fescue manifest clinical symptoms of fescue toxicosis (“summer slump”), such as decreased body weight (BW) gain (1–3), reduced feed intake (4, 5), lowered milk production (6), retained winter hair coat during the summer (7), increased body temperature (6), and increased respiration rate (4).

The consumption of endophyte-infected tall fescue also is known to alter the blood parameter profiles of cattle and other livestock, with decreased plasma prolactin being the most commonly observed indicator of fescue toxicosis (8). Previously, we reported (9) the effects of fescue toxicosis on developing Angus-cross steer growth, carcass, hepatic mRNA, and protein expression profiles of selected serum proteins, and blood clinical and chemical profiles that were induced by whole-summer-long grazing of forages containing either high or low amounts of endophyte-infected tall fescue. Therefore, the primary objectives of the current research were to (a) characterize the temporal changes in blood clinical and chemical profiles of steers grazing forages containing either high or low amounts of endophyte-infected tall fescue over the course of the summer and then (b) evaluate the potential relationships between serum prolactin and measured blood analytes.

## Materials and Methods

### Animals and Experimental Periods

All experimental procedures were approved by the University of Kentucky Institutional Animal Care and Use Committee (protocol no. 01012A2006). Nineteen predominately Angus crossbred beef steers were randomly allotted (day 0) to graze low-toxic endophyte tall fescue-mixed grass (LE, *n* = 9, 5.7 ha; BW = 266 ± 10.9 kg) or a high-toxic endophyte-infected tall fescue (HE, *n* = 10, 5.7 ha; BW = 267 ± 14.5 kg) for 85 days. Pastures were within 500 m of each other and are part of the University of Kentucky Agricultural Research Center, located in Woodford County, KY, USA. All steers had *ad libitum* access to fresh water and mineral supplement (Ca min. 13.0 – max. 15.0%; *P* 6.2%; NaCl min. 17.0 – max. 19.5%; Mg 3.0%; S 1.0%; K 0.8%; Zn 2300 μg/g; Mn 2200 μg/g; Cu 1450 μg/g; I 45 μg/g; Co 15 μg/g; Se 29 μg/g; Vit. A 661 IU/g; Vit. E 0.276 IU/g; as-fed). Shrunk (denied access to feed and water for 14 h) BW were determined on day 0 and 86 to determine overall experiment average daily gain (ADG). Full BW were taken on day 36 and 57 and shrunk BW calculated based on the mean percentage shrink of 4.04% from weight measurements taken on day 0 and 85. Experimental periods were as per weigh days: period 1 = day 0–36, period 2 = day 37–58, and period 3 = day 59–85.

### Pasture Sampling and Analysis

On day 37, 59, and 88 of the study, leaf blades suitable for grazing were detached from each fescue plant in pasture for ergot alkaloid (ergovaline, ergovalinine, lysergic acid, and isolysergic acid) determination, and proximate analysis (9). Briefly, samples were obtained systematically from approximately 30 sites in each pasture, using a knife to cut the forage at approximately 2 cm above soil level. Samples were immediately placed into individual plastic bags, and then stored on ice during transportation to our laboratory. All samples were frozen and stored at −20°C. Analysis of ergot alkaloids was performed, as previously described (10), and isolysergic acid was quantified with a lysergic acid standard. Proximate analysis and mineral content were determined by a commercial laboratory (Dairy One Forage Lab, Ithaca, NY, USA).

### Blood Collection and Analyses

Jugular venous blood samples were collected by venipuncture on day 36, 58, and 85. For plasma, 16 mL of blood was collected in EDTA-containing (0.9375 mg/mL) blood collection tubes (Becton, Dickinson and Company, Franklin, Lakes, NJ, USA). For serum, 16 mL of blood was collected in serum blood collection tubes without an anticoagulant. For whole blood, 2 mL of blood was collected in EDTA-containing (2.7 mg/mL) blood collection tubes (Becton Dickerson). Plasma and sera were recovered by refrigerated centrifugation at 3 000 × *g* for 10 min at 4°C and stored at −80°C. Plasma samples were analyzed for ammonia-N by modifications of the l-Glu dehydrogenase enzyme assay (11) using a Konelab 20XTi analyzer (Thermo Electron Corp., Finland). Serum prolactin analysis (12) was conducted (Dr. F. N. Schrick Laboratory, Johnson Animal Research and Teaching Unit, University of Tennessee-Knoxville). All other serum enzymes and analytes, and blood cell types, were analyzed by the American Association for Veterinary Laboratory Diagnosticians approved – University of Kentucky Livestock Disease Diagnostic Center (Lexington, KY, USA). For serum analytes, activities of alkaline phosphatase (ALP), E.C. 3.1.3.1; alanine transaminase (ALT), E. C. 2.6.1.2; aspartate transaminase (AST), E. C. 2.6.1.1; γ-glutamyltransferase, E. C. 2.3.22; creatine kinase, E. C. 2.7.3.2; and lactate dehydrogenase (LDH), E. C. 1.1.1.27 were determined as per the manufacturer of the reagent kits (Alfa Wassermann, Diagnostic Technologies, West Caldwell) using a VET-EX Chemical Analyzer (Alfa-Wassermann), as were the other serum analytes. The concentration of red blood cells (RBCs), white blood cells, packed cell volume, and hemoglobin in whole blood was determined using a Hemavet HV 950S cell analyzer (Drew Scientific Inc., Miami Lakes, FL, USA). The whole blood concentration of neutrophils, lymphocytes, monocytes, and eosinophils were determined by manual identification and counting of cells (13).

### Statistical Methods

Data are presented as least square means (±SEM). Individual steers were the experimental units. The effect of grazing HE vs. LE pastures on all measured experimental parameters was evaluated by ANOVA, using the MIXED procedure of SAS (v 8.01, SAS Inst. Inc., Cary, NC, USA). The statistical model used fescue toxicosis, the experimental period, and their interaction as fixed effects. Class variables were fescue toxicosis and steer, with steer included in the random statement. The Kenward–Roger adjustment was used to calculate the denominator df (14). Linear and quadratic non-orthogonal polynomial contrasts were used to characterize the effect of treatment over time using the imi procedure of SAS. Partial correlations between prolactin concentrations and serum analytes were determined by using ANOVA (PROC GLM) by using the MANOVA/PRINTE statement of SAS. For all data, significance was declared when *P* ≤ 0.05 and tendency to differ was declared when 0.10 ≥ *P* > 0.05.

## Results

### Nutrient Profiles of HE and LE Forages

Across-periods, the % compositions of DM, ADF, and crude fat did not differ (*P* ≥ 0.205) between HE and LE pastures (Table 1). However, the HE forage contained less (*P* ≤ 0.001) lignin (24%) and NDF (4.3%), and more (*P* ≤ 0.001) TDN (3.8%), CP (7.6%), and ash (7.3%) than did LE.

**Table 1:** **Proximate analysis of low endophyte (LE) and high endophyte (HE) pasture samples^a^**.

Item (%)	Pasture treatment			*P*-value		
	LE	HE	SEM	TRT	Period	TRT by period
**Across-periods**						
DM	25.5	26.1	0.46	0.356	<0.001	0.273
CP	17.10	18.40	0.195	0.001	0.002	<0.001
TDN	58.56	60.78	0.377	0.001	0.001	0.093
ADF	32.12	31.83	0.370	0.591	0.782	0.069
NDF	60.41	57.82	0.387	0.001	0.008	0.004
Crude fat	3.46	3.61	0.082	0.205	0.006	0.179
Lignin	6.23	4.76	0.247	0.001	0.012	0.215
Ash	8.20	8.80	0.09	0.001	0.077	0.007
**Within-periods**						
DM
Period 1	28.5	27.8	0.79	0.544		
Period 2	26.8	28.8	0.79	0.100		
Period 3	21.1	21.6	0.79	0.623		
CP
Period 1	16.50	20.83	0.34	<0.001		
Period 2	16.70	17.93	0.34	0.024		
Period 3	18.10	16.43	0.34	0.004		
TDN
Period 1	56.00	60.00	0.653	0.001		
Period 2	58.67	60.33	0.653	0.096		
Period 3	61.00	62.00	0.653	0.300		
ADF
Period 1	32.87	30.93	0.641	0.054		
Period 2	32.40	32.07	0.641	0.720		
Period 3	31.10	32.50	0.641	0.149		
NDF
Period 1	62.83	57.47	0.670	<0.001		
Period 2	60.87	58.17	0.670	0.015		
Period 3	57.53	57.83	0.670	0.757		
Crude fat
Period 1	3.07	3.50	0.142	0.052		
Period 2	3.77	3.93	0.142	0.423		
Period 3	3.53	3.40	0.142	0.520		
Lignin
Period 1	7.33	5.03	0.428	0.003		
Period 2	6.37	4.93	0.428	0.035		
Period 3	5.00	4.30	0.428	0.270		
Ash
Period 1	7.9	9.1	0.16	0.001		
Period 2	8.4	9.1	0.16	0.008		
Period 3	8.4	8.3	0.16	0.864		

*^a^Proximate analysis values are presented on a DM basis. Samples were obtained systematically from approximately 30 sites in each pasture, using a knife to cut the forage at approximately 2 cm above soil level. Data are presented as least square means (±SEM)*.

### Ergot Alkaloid Profiles of Forages

Across-period analysis of ergot alkaloid levels between the two forages revealed that the HE steers were exposed to 19 and 25 times more (*P* ≤ 0.002) ergovaline/ergovalinine and lysergic acid/isolysergic acid, respectively, than LE steers (Table 2). No period or treatment by period interactions was observed for lysergic and isolysergic acids indicating that the differences in the concentration between HE and LE forages were similar throughout the trial. For ergovaline and ergovalinine, however, period (*P* ≤ 0.004) and treatment by period (*P* ≤ 0.008) were observed, perhaps reflecting the exceptionally low values for LE forages during period 1.

**Table 2:** **Alkaloid analysis of composited low endophyte (LE)- and high endophyte (HE)-infected forage samples^a^**.

Alkaloid (μg/g)	Pasture treatments			*P*-value		
	LE	HE	SEM	TRT	Period	TRT by period
**Across-periods**						
Ergovaline	0.018	0.276	0.016	<0.001	0.004	0.008
Ergovalinine	0.004	0.152	0.005	<0.001	<0.001	<0.001
Lysergic acid	0.002	0.064	0.011	0.002	0.731	0.908
Isolysergic acid	0.007	0.156	0.019	0.001	0.821	0.893
**Within-periods**						
Ergovaline
Period 1	<0.001	0.260	0.028	<0.001		
Period 2	0.023	0.173	0.028	0.002		
Period 3	0.030	0.393	0.028	<0.001		
Ergovalinine
Period 1	<0.001	0.087	0.008	<0.001		
Period 2	0.010	0.100	0.008	<0.001		
Period 3	0.003	0.270	0.008	<0.001		
Lysergic acid
Period 1	<0.001	0.053	0.019	0.071		
Period 2	0.007	0.077	0.019	0.023		
Period 3	<0.001	0.063	0.019	0.037		
Isolysergic acid
Period 1	<0.001	0.140	0.032	0.010		
Period 2	0.020	0.160	0.032	0.010		
Period 3	<0.001	0.167	0.032	0.032		

*^a^Across- and within-periods proximate analysis values are presented on a DM basis. Samples were obtained systematically from approximately 30 sites in each pasture, using a knife to cut the forage at approximately 2 cm above soil level. Data are presented as least square means (±SEM)*.

### Steer Growth

Across-periods, BW tended to be lower (*P* = 0.097), and ADG was lower (*P* = 0.014), for HE vs. LE steers (Table 3). For BW, a period effect was found (*P* ≤ 0.001) reflecting the greater growth of HE and LE steers during period 3. Similarly, the ADG for period 3 appeared greater for both groups of steers than for the other periods. Moreover, a treatment by period interaction was found (*P* = 0.026) for ADG. That is, although the ADG of LE and HE steers did not differ (*P* ≥ 0.595) in periods 2 and 3, during period 1 LE steers achieved an ADG of 0.4 kg, whereas HE steers lost (*P* = 0.003) BW (0.05 kg/day). Overall, from day 0 to 85, the ADG of HE steers was 0.18 kg more (*P* = 0.014) than for LE steers.

**Table 3:** **Body weight and ADG gain of steers grazing forages containing low endophyte (LE)- and high endophyte (HE)-infected forages for 85 days**.

Item	*P*-value			
	TRT	Period	TRT by period	
Across-periods
BW (kg)^a^	0.097	<0.001	0.861	
ADG (kg)	0.014	<0.001	0.026	
Within-periods

**Item**	**Pasture treatment^b^**			
	**LE**	**HE**	**SEM**	***P*-value**

No. of steers	9	10		
BW (kg)^a^
Day 36	280	265	6.1	0.091
Day 58	287	273	6.1	0.094
Day 85	314	301	6.1	0.132
ADG (kg)
Period 1	0.40	−0.05	0.10	0.003
Period 2	0.26	0.27	0.10	0.971
Period 3	1.3	1.4	0.10	0.595
Overall	0.58	0.40	0.05	0.014

*^a^BW are calculated, or measured shrunk weights, as described in text*.

*^b^Steers LE (ergovaline and ergovalinine 0.02 μg; lysergic acids 0.01 μg) or HE (ergovaline and ergovalinine 0.52 μg; lysergic acids 0.22 μg) endophyte-infected tall fescue pastures*.

### Prolactin

Across all three periods, serum prolactin levels of HE steers (*P* ≤ 0.001) were 15% that of LE steers (Table 4). Steers grazing HE forage had serum prolactin values that were 14, 19, and 10% that of LE steers in period 1, 2, and 3, respectively. The level of prolactin decreased in a quadratic manner (*P* ≤ 0.012) over time for both LE and HE steers (Table 5), with the concentration of prolactin in the steers grazing LE pasture decreasing 58.3 ng/mL from periods 1 to 3 (Table 4). Steers grazing HE pasture exhibited a smaller decrease in prolactin (9.9 ng/mL) across-periods, likely due to the fact that HE steer prolactin levels had already been suppressed severely in period 1. A treatment by period interaction likely reflects this time-dependent differential decrease in serum prolactin values.

**Table 4:** **Serum and plasma analytes of steers grazing low endophyte (LE)- or high endophyte (HE)-infected forages^a^**.

Item^b^	Pasture treatment			*P*-value			Reference range
	LE	HE	SEM^c^	TRT	Period	TRT by period	
**Across-periods**							
Prolactin (ng/mL)	70.8	10.8	3.09	<0.001	<0.001	<0.001	–
ALP (U/L)	111.3	82.4	7.08	0.079	<0.001	0.009	100.0–500.0^d^
ALT (U/L)	30.4	27.5	0.96	0.040	<0.001	0.149	11–40^e^
AST (U/L)	68.1	61.9	2.53	0.092	0.495	0.106	0–160^e^
AST/ALT ratio	2.30	2.33	0.115	0.823	0.007	0.961	–
Ammonia^f^ (mM)	0.059	0.070	0.005	0.077	<0.001	0.052	–
Blood urea nitrogen (mg/100 mL)	17.0	17.7	0.60	0.459	<0.001	0.680	5.0–27.0^d^
Albumin (g/100 mL)	3.68	3.48	0.034	0.009	<0.001	0.860	2.30–3.70^d^
Globulin (g/100 mL)	3.27	3.34	0.062	0.556	<0.001	0.719	3.0–3.5^d^
Albumin/globulin ratio	1.17	1.07	0.025	0.037	<0.001	0.731	0.8–1.00^d^
γ-Glutamyltransferase (U/L)	12.1	11.3	0.43	0.276	<0.001	0.735	2.0–20.0^d^
Total bilirubin (mg/100 mL)	0.2	0.2	0.01	0.453	0.166	0.166	0.0–0.5^d^
Total protein (g/100 mL)	6.95	6.82	0.067	0.318	<0.001	0.569	6.50–7.50^d^
Creatinine (mg/100 mL)	1.29	1.28	0.049	0.857	<0.001	0.578	1.00–2.00^d^
Blood urea nitrogen:creatinine	13.33	13.90	0.387	0.305	0.424	0.489	40–100^d^
Creatine kinase (U/L)	165.0	126.0	8.70	0.032	<0.001	0.010	100.0–650^d^
Glucose (mg/100 mL)	71	65	1.6	0.068	0.001	0.530	40–100^d^
LDH (U/L)	1037	870	34.1	0.007	0.322	0.224	692–1445^e^
Triglycerides (g/100 mL)	30	26	1.4	0.212	0.002	0.003	–
Cholesterol (mg/100 mL)	95	69	2.2	<0.001	<0.001	0.354	62–193^d^
**Within-periods**							
Prolactin (ng/mL)
Period 1	94.3	13.5	3.94	<0.001			
Period 2	82.1	15.2	3.94	<0.001			
Period 3	36.0	3.6	3.94	<0.001			
ALP (U/L)
Period 1	93.0	71.6	12.27	0.218			
Period 2	81.2	66.5	12.27	0.393			
Period 3	159.8	109.1	12.27	0.006			
ALT (U/L)
Period 1	33.3	30.1	1.41	0.102			
Period 2	25.4	25.1	1.41	0.860			
Period 3	32.6	27.3	1.41	0.009			
AST (U/L)
Period 1	66.8	63.7	2.94	0.454			
Period 2	66.3	61.3	2.94	0.225			
Period 3	71.2	60.6	2.94	0.014			
AST/ALT ratio
Period 1	2.05	2.14	0.180	0.723			
Period 2	2.63	2.63	0.180	0.995			
Period 3	2.21	2.23	0.180	0.934			
Ammonia^e^ (mM)
Period 1	0.078	0.078	0.0055	0.940			
Period 2	0.053	0.071	0.0055	0.026			
Period 3	0.045	0.062	0.0055	0.035			
Blood urea nitrogen (mg/100 mL)
Period 1	18.8	19.8	0.73	0.314			
Period 2	16.1	16.8	0.73	0.496			
Period 3	16.2	16.4	0.73	0.860			
Albumin (g/100 mL)
Period 1	3.94	3.76	0.060	0.032			
Period 2	3.77	3.54	0.060	0.009			
Period 3	3.33	3.14	0.060	0.025			
Globulin (g/100 mL)
Period 1	2.93	3.08	0.107	0.327			
Period 2	3.04	3.07	0.107	0.864			
Period 3	3.83	3.86	0.107	0.858			
Albumin/globulin
Period 1	1.37	1.24	0.043	0.039			
Period 2	1.24	1.15	0.043	0.119			
Period 3	0.90	0.83	0.043	0.245			
γ-Glutamyltransferase (U/L)
Period 1	12.0	11.6	0.75	0.699			
Period 2	13.8	13.0	0.75	0.454			
Period 3	10.6	9.2	0.75	0.195			
Total bilirubin (mg/100 mL)
Period 1	0.20	0.24	0.01	0.055			
Period 2	0.23	0.22	0.01	0.515			
Period 3	0.20	0.20	0.01	1.000			
Total protein (g/100 mL)
Period 1	6.88	6.84	0.117	0.816			
Period 2	6.81	6.61	0.117	0.220			
Period 3	7.17	7.00	0.117	0.308			
Creatinine (mg/100 mL)
Period 1	1.44	1.39	0.057	0.496			
Period 2	1.19	1.21	0.057	0.791			
Period 3	1.23	1.23	0.057	0.967			
Blood urea nitrogen:creatinine
Period 1	13.17	14.24	0.494	0.126			
Period 2	13.60	14.00	0.494	0.554			
Period 3	13.23	13.45	0.494	0.750			
Creatine kinase (U/L)
Period 1	144.0	119.3	15.06	0.242			
Period 2	127.7	113.8	15.06	0.509			
Period 3	223.4	144.8	15.06	0.001			
Glucose (mg/100 mL)
Period 1	69	64	2.8	0.181			
Period 2	66	63	2.8	0.455			
Period 3	78	70	2.8	0.040			
LDH (U/L)
Period 1	952	889	59.0	0.440			
Period 2	1095	901	59.0	0.022			
Period 3	1063	821	59.0	0.004			
Triglycerides (g/100 mL)
Period 1	33	24	2.4	0.017			
Period 2	27	23	2.4	0.204			
Period 3	30	31	2.4	0.695			
Cholesterol (mg/100 mL)
Period 1	95	71	3.75	<0.001			
Period 2	83	59	3.75	<0.001			
Period 3	107	76	3.75	<0.001			

*^a^Data are presented as least squares means (±SEM) of LE (*n* = 9) and HE (*n* = 10) treatments*.

*^b^ALP, alkaline phosphatase; ALT, alanine aminotransferase; AST, aspartate aminotransferase; LDH, lactate dehydrogenase*.

*^c^Most conservative error of the mean*.

*^d^Taken from the University of Kentucky Livestock Disease Diagnostic Laboratory (Lexington, KY, USA)*.

*^e^Taken from Kaneko et al. (15)*.

*^f^Plasma*.

**Table 5:** **Linear vs. quadratic time effects on serum and plasma analytes of steers grazing low endophyte (LE)- or high endophyte (HE)-infected forages^a^^,^^b^**.

Item^b^	LE		HE	
	Linear	Quadratic	Linear	Quadratic
Prolactin	<0.001	0.010	0.001	0.012
ALP	0.002	0.024	0.009	0.080
ALT	0.863	<0.001	0.194	0.035
AST	0.204	0.432	0.031	0.383
AST/ALT ratio	0.341	<0.001	0.822	0.066
Ammonia	<0.001	0.0960	0.001	0.954
Blood urea nitrogen	<0.001	0.002	0.001	0.050
Albumin	<0.001	0.180	<0.001	0.425
Globulin	<0.001	0.033	<0.001	0.008
Albumin/globulin ratio	<0.001	0.112	<0.001	0.071
γ-Glutamyltransferase	0.110	0.008	0.023	0.013
Total bilirubin	0.907	0.057	0.069	0.912
Total protein	0.062	0.156	0.283	0.044
Creatinine	0.001	0.002	0.011	0.032
Blood urea nitrogen:creatinine	0.952	0.335	0.170	0.809
Creatine kinase	0.010	0.055	0.003	0.024
Glucose	0.031	0.036	0.141	0.255
LDH	0.343	0.337	0.151	0.308
Triglycerides	0.482	0.253	0.015	0.075
Cholesterol	0.056	0.005	0.124	0.001

*^a^Data are *P*-values associated with linear and quadratic non-orthogonal polynomial contrasts used to characterize the effect of treatment over time on concentrations of blood analytes*.

*^b^ALP, alkaline phosphatase; ALT, alanine aminotransferase; AST, aspartate aminotransferase; LDH, lactate dehydrogenase*.

### Biochemical and Clinical Blood Profiles

As shown in Table 4, the mean value across-periods for 9 of the biochemical and clinical blood profile measurements did not change (*P* ≥ 0.212), whereas ALP, ALT, AST, ammonia, albumin, albumin:globulin ratio, creatine kinase, glucose, LDH, and cholesterol were influenced (*P* < 0.10) by grazing treatment. The trend across time for each biochemical and blood parameter was also determined for each treatment group (Table 5).

Serum ALP activity for HE steers tended (*P* = 0.079) to exhibit 26% less activity than LE steers (Table 4). In period 3, ALP was reduced (*P* = 0.006) by 32% for HE steers. Furthermore, there was a significant (*P* < 0.001) period interaction (Table 4), which showed quadratic trends (*P* = 0.024) (Table 5) for ALP for LE steers that decreased 13% from period 1 to 2 and increased 97% from period 2 to 3. For HE steers, there was also a quadratic trend (*P* = 0.080) (Table 5), which reduced ALP by 7.1% from periods 1 to 2 and increased 64% from periods 2 to 3. The period by treatment interaction (*P* = 0.009) most likely was due to the large increase in ALP by the LE steers from periods 2 to 3 (Table 4).

Across-periods, ALT activity was reduced (*P* = 0.040) by 9.5% in HE consuming steers compared to the LE consuming steers (Table 4). In period 3, ALT also was found to be depressed 16% (*P* = 0.009) in HE steers (Table 4). The period effect (*P* = 0.001) (Table 5) can be explained by the quadratic response to time (*P* ≤ 0.035) (Table 5) for both treatments, with activity depressed during period 2 compared to periods 1 and 3. ALT activity for steers consuming LE pastures decreased by 24% from periods 1 to 2 and increased by 28% from periods 2 to 3, whereas ALT activity for HE consuming steers decreased by 17% from periods 1 to 2 and increased by 8.8% from period 2 to 3 (Table 4).

Across-periods, HE steer serum AST activity decreased (*P* = 0.092) 9.1% (Table 4). Within-periods, AST was decreased (*P* = 0.014) by 15% in HE vs. LE steers in period 3 and demonstrated no period or period by treatment interaction (Table 4). Across-periods, there was a linear decrease (*P* = 0.031) (Table 5) of 4.9% for HE steers across-periods but no trend for LE steers. For AST, the mean differed but there was no period or treatment by period interaction unlike prolactin and ALT, despite the fact that LE appeared to increase and HE to decrease (Table 4).

The serum albumin content of HE steers was 5% lower (*P* ≤ 0.009) than LE steers across-periods (Table 4). Within-periods 1, 2, and 3, serum albumin of the HE steers was 4.6, 6.1, and 5.7% less (*P* ≤ 0.032), respectively, than that of LE steers. The period effect could be explained by the fact that LE and HE steers decreased linearly (*P* < 0.001) (Table 5) in a parallel manner by 15 and 16%, respectively (Table 4). Additionally, the albumin:globulin ratio in HE steers was decreased (*P* = 0.037) by 8.5% across-periods when compared to LE steers (Table 4). In period 1, the albumin:globulin ratio was decreased by 9.5% in HE when compared to LE steers. The period effect (*P* < 0.001) was evident in that both treatment groups decreased over time with LE steers decreasing linearly (*P* < 0.001), whereas HE steers displayed a quadratic (*P* = 0.071) decrease across-periods (Table 5).

Across treatments, the creatine kinase activity that was 24% less (*P* = 0.032) in HE vs. LE steers (Table 4). In period 3, creatine kinase activity in HE steers was (*P* = 0.001) 35% less than that of LE steers. From periods 1 to 2, LE and HE steers decrease in a parallel manner. Nonetheless, from periods 2 to 3, LE increased by 75% while HE consuming steers creatine kinase activity increased by only 27%, likely reflecting the period and period by treatment interaction.

Glucose concentrations were 8.5% lower (*P* = 0.068) in HE vs. LE steers across-periods (Table 4). In period 3, HE steers exhibited (*P* = 0.040) a 10% lower concentration of glucose. The period effect could be explained by a slight parallel decrease in serum glucose from periods 1 to 2 for LE and HE steers of 4.3 and 1.6% and increased from periods 2 to 3 by 18 and 11%, respectively. LE steers showed a quadratic response (*P* = 0.036), whereas HE steers demonstrated no linear or quadratic response (Table 5).

Across-periods, LDH activity was reduced (*P* = 0.007) 16% of HE steers vs. LE steers (Table 4), with LDH activity decreased (*P* ≤ 0.022) by 18 and 23%, respectively, in periods 2 and 3. Similar to AST, the means that for LDH differed but there was no period or treatment by period interaction, even though the LE steer mean appeared to increase and HE steer mean decreased across-periods (Table 5).

There was no significant change (*P* = 0.212) in triglycerides across-periods between the two treatment groups (Table 4). Nonetheless, serum triglyceride concentration was reduced (*P* = 0.017) by 27% during period 1 in HE steers. A treatment by period interaction (*P* = 0.003) reflects a decreased triglyceride concentration from periods 1 to 2 for HE steers and increased concentrations from periods 2 to 3.

Across-periods, HE steers exhibited a 27% decrease (*P* < 0.001) in cholesterol as compared to LE steers (Table 4), with serum cholesterol concentrations that were 25, 29, and 29% less (*P* < 0.001) that of LE steers in periods 1, 2, and 3, respectively. The period effect (*P* < 0.001) can be explained by the fact that both treatments responded in a parallel quadratic manner (*P* ≤ 0.005) (Table 5). From periods 1 to 2, there was a decrease of 13 and 17%; and from periods 2 to 3, there was an increase of 29 and 29% for LE and HE, respectively (Table 4), which was consistent with the lack of a treatment by period interaction (*P* = 0.354).

In contrast to other biochemical parameters, ammonia plasma concentrations tended (*P* = 0.077) to be increased in HE steers (Table 4). Across-periods, ammonia levels were 19% greater for HE steers. Within-periods 2 and 3, the ammonia concentration of HE steers was 34 and 38% greater (*P* ≤ 0.035), respectively, than that of LE steers. The period effect (*P* < 0.001) could be explained by the fact that over time both treatment groups exhibited a decrease. The treatment by time interaction (*P* = 0.052) was apparent as LE steers exhibited a quadratic trend (*P* = 0.096) (Table 5), decreasing 32% from periods 1 to 2 and then 15% from periods 2 to 3. Nonetheless, the HE steers exhibited a linear (*P* = 0.001) decrease of 21% in plasma ammonia (Table 5).

### Blood Cell Types

Across-periods, the amount of RBCs was increased (*P* = 0.049) 6.9% in HE vs. LE steers (Table 6). By contrast, other types of blood cells were not affected (*P* ≥ 0.228). Within-periods, RBC concentrations of HE steers were increased (*P* ≤ 0.10) by 6.3, 7.2, and 7.1% that of LE steers in periods 1, 2, and 3, respectively. The period effect (*P* = 0.092) was due to the fact that both LE and HE steers displayed a decrease in RBC during period 2.

**Table 6:** **Blood cell types of steers grazing low endophyte (LE)- or high endophyte (HE)-infected forages^a^**.

Item	Pasture treatment			*P*-value			Reference range
	LE	HE	SEM^b^	TRT	Period	TRT × period
**Across-periods**							
RBC^c^ (1 × 10^6^/μL)	8.43	9.01	0.134	0.049	0.092	0.974	5.0–10^e^
Hemoglobin (g/dL)	10.8	10.9	0.17	0.811	0.001	0.539	8.0–15^e^
Packed cell volume (%)	30.1	30.5	0.50	0.701	<0.001	0.783	24–46^e^
WBC^d^ (1 × 10^3^/μL)	9.26	8.89	0.606	0.663	0.010	0.296	4.0–12.0^e^
Neutrophils (1 × 10^3^/μL)	3.13	3.18	0.331	0.917	0.004	0.900	0.06–4.00^f^
Lymphocytes (1 × 10^3^/μL)	5.65	5.23	0.373	0.430	0.044	0.240	2.5–7.5^f^
Monocytes (1 × 10^3^/μL)	0.43	0.35	0.046	0.228	<0.001	0.243	0.0–0.9^f^
Eosinophils (1 × 10^3^/μL)	0.22	0.17	0.046	0.373	0.183	0.087	0.0–2.4^f^
**Within-periods**							
RBC^c^ (1 × 10^6^/μL)							
Period 1	8.57	9.11	0.231	0.100			
Period 2	8.24	8.83	0.231	0.077			
Period 3	8.49	9.09	0.231	0.067			
Hemoglobin (g/dL)							
Period 1	10.8	11.1	0.29	0.472			
Period 2	10.5	10.5	0.29	0.989			
Period 3	11.1	11.1	0.29	0.945			
Packed cell volume (%)							
Period 1	29.5	30.2	0.87	0.580			
Period 2	28.6	29.2	0.87	0.657			
Period 3	32.3	32.3	0.87	0.973			
WBC^d^ (1 × 10^3^/μL)							
Period 1	10.43	9.12	0.743	0.209			
Period 2	8.11	8.19	0.743	0.942			
Period 3	9.23	9.35	0.743	0.904			
Neutrophils (1 × 10^3^/μL)							
Period 1	3.14	3.03	0.411	0.857			
Period 2	2.62	2.72	0.411	0.864			
Period 3	3.65	3.80	0.411	0.792			
Lymphocytes (1 × 10^3^/μL)							
Period 1	6.58	5.42	0.483	0.090			
Period 2	5.11	5.09	0.483	0.976			
Period 3	5.25	5.18	0.507	0.924			
Monocytes (1 × 10^3^/μL)							
Period 1	0.64	0.53	0.066	0.263			
Period 2	0.27	0.31	0.066	0.723			
Period 3	0.38	0.21	0.070	0.081			
Eosinophils (1 × 10^3^/μL)							
Period 1	0.38	0.17	0.087	0.056			
Period 2	0.18	0.13	0.077	0.625			
Period 3	0.11	0.21	0.065	0.245			

*^a^Data are presented as least squares means (±SEM) of LE (*n* = 9) and HE (*n* = 10) treatments*.

*^b^Most conservative error of the mean*.

*^c^Red blood cells*.

*^d^White blood cells*.

*^e^Taken from the University of Kentucky Livestock Disease Diagnostic Laboratory (Lexington, KY, USA)*.

*^f^Taken from Duncan et al. (16)*.

Period affected blood cell abundance (*P* ≤ 0.044) with the exception of eosinophils, which demonstrated a treatment by period interaction (*P* = 0.087) (Table 6). This interaction appears to reflect the linear (*P* = 0.016) (Table 7) decrease with time of LE eosinophils, whereas HE steer eosinophils appeared to increase in period 3. Lymphocytes were decreased (*P* = 0.090) in HE steers by 18% in period 1, and monocytes were decreased (*P* = 0.070) by 45% in period 3. The LE steers show a linear (*P* = 0.060) (Table 7) decline across-period for lymphocytes and the means of the HE steers appears to parallel that of the LE steers. The decline across-periods reflects a significant (*P* = 0.044) period effect for lymphocytes, but the parallel pattern did not allow for a treatment by period interaction (*P* = 0.240). The LE steers displayed a quadratic (*P* = 0.016) (Table 7) response for monocytes across-period with period 2 being depressed the most. The HE steers exhibited a linear (*P* ≤ 0.001) decline for monocytes across-periods. The overall decline in monocytes across time was enough to account for a period effect, but the differences in the trends of each treatment group was not enough to allow for a significant treatment by period interaction (*P* = 0.243) (Table 6).

**Table 7:** **Linear vs. quadratic time effects on blood cell type of steers grazing low endophyte (LE)- or high endophyte (HE)-infected forages^a^**.

Item	LE		HE	
	Linear	Quadratic	Linear	Quadratic
RBC^b^	0.808	0.195	0.994	0.388
Hemoglobin	0.347	0.124	0.979	0.119
Packed cell volume	0.008	0.025	0.090	0.095
WBC^c^	0.224	0.029	0.617	0.059
Neutrophils	0.260	0.093	0.029	0.039
Lymphocytes	0.060	0.117	0.639	0.581
Monocytes	0.033	0.016	<0.001	0.161
Eosinophils	0.016	0.307	0.599	0.473

*^a^Data are *P*-values associated with linear and quadratic non-orthogonal polynomial contrasts used to characterize the effect of treatment over time on blood cell type*.

*^b^Red blood cells*.

*^c^White blood cells*.

### Correlation of Serum Prolactin with Blood Analytes

Correlation analyses were performed to determine if a relationship existed between prolactin levels and serum and plasma (Table 8) and blood cell types (Table 9). Across treatments, the relative abundance of prolactin was weakly (0.321 ≥ *r* ≥ 0.236) correlated with ALT (*P* = 0.022), blood urea nitrogen (*P* = 0.092), triglycerides (*P* = 0.021), and hemoglobin *(P* = 0.043). Within treatments, correlation coefficients for ALT (*r* = 0.424), blood urea nitrogen (*r* = 0.405), and hemoglobin (*r* = 0.536) were significant (*P* ≤ 0.045) and stronger only for LE steers, whereas the correlation coefficient for triglycerides (*r* = 0.388) was significant (*P* = 0.042) and stronger only for HE steers. Within treatment prolactin by analyte correlation analysis also revealed a significant (*P* = 0.021) moderately strong correlation (*r* = 0.433) between prolactin and creatinine in HE but not LE steers, whereas correlations between prolactin and blood urea nitrogen:creatinine (*r* = 0.377; *P* = 0.064) and packed cell volume (*r* = 0.375; *P* = 0.065) were identified in LE but not HE steers.

**Table 8:** **Correlation of serum and plasma analytes with serum prolactin of steers grazing low endophyte (LE)- or high endophyte (HE)-infected forages**.

Item^d^	Overall^a^		LE^b^		HE^c^	
	
	Coefficient	*P*-value	Coefficient	*P*-value	Coefficient	*P*-value
ALP	0.176	0.212	0.155	0.4617	0.272	0.161
ALT	0.317	0.022	0.424	0.035	0.268	0.168
AST	0.161	0.256	0.154	0.462	0.202	0.302
AST/ALT ratio	−0.119	0.398	−0.144	0.492	−0.140	0.477
Ammonia	−0.146	0.301	−0.231	0.267	0.088	0.657
Blood urea nitrogen	0.236	0.092	0.405	0.045	0.204	0.297
Albumin	−0.111	0.434	−0.237	0.254	0.116	0.558
Globulin	0.092	0.517	0.214	0.305	−0.149	0.449
Albumin/globulin ratio	−0.159	0.262	−0.342	0.094	0.215	0.272
γ-Glutamyltransferase	0.100	0.479	0.240	0.247	−0.136	0.490
Total bilirubin	0.122	0.389	0.220	0.290	−0.024	0.904
Total protein	0.028	0.845	0.083	0.695	−0.074	0.709
Creatinine	0.110	0.438	−0.019	0.927	0.433	0.021
Blood urea nitrogen:creatinine	0.121	0.392	0.377	0.064	−0.237	0.226
Creatine kinase	0.023	0.870	0.014	0.948	0.109	0.614
Glucose	−0.057	0.689	−0.145	0.490	0.120	0.542
LDH	0.018	0.898	0.046	0.827	−0.120	0.542
Triglycerides	0.321	0.021	0.314	0.126	0.388	0.042
Cholesterol	0.046	0.748	0.054	0.799	0.019	0.923

*^a^(LE + HE) analytes vs. (LE + HE) prolactin*.

*^b^LE analytes vs. LE prolactin*.

*^c^HE analytes vs. HE prolactin*.

*^d^ALP, alkaline phosphatase; ALT, alanine aminotransferase; AST, aspartate aminotransferase; LDH, lactate dehydrogenase*.

**Table 9:** **Correlation of blood cell type with serum prolactin of steers grazing low endophyte (LE)- or high endophyte (HE)-infected forages**.

Item	Overall^a^		LE^b^		HE^c^	
	Coefficient	*P*-value	Coefficient	*P*-value	Coefficient	*P*-value
RBC^d^	−0.144	0.307	−0.322	0.116	0.039	0.842
Hemoglobin	0.282	0.043	0.536	0.006	0.013	0.949
Packed cell volume	0.197	0.162	0.375	0.065	−0.005	0.982
WBC^e^	0.112	0.430	0.099	0.639	0.177	0.368
Neutrophils	0.193	0.170	0.277	0.180	0.127	0.519
Lymphocytes	−0.033	0.818	−0.103	0.633	0.151	0.445
Monocytes	0.062	0.669	0.055	0.799	0.094	0.643
Eosinophils	−0.078	0.649	−0.142	0.627	−0.051	0.816

*^a^(LE + HE) analytes vs. (LE + HE) prolactin*.

*^b^LE analytes vs. LE prolactin*.

*^c^HE analytes vs. HE prolactin*.

*^d^Red blood cells*.

*^e^White blood cells*.

## Discussion

### Trial-Long (Across All Periods) Responses

The primary objectives of this research were to characterize the temporal changes in blood clinical and chemical profiles of beef steers grazing forages containing either high or low amounts of endophyte-infected tall fescue over the course of the summer. Forage analysis revealed that the high alkaloid concentration in HE vs. LE pastures was maintained throughout the trial. Of the 19 blood analytes and 8 blood cell type HE vs. LE treatment responses measured across all 3 experimental periods of the trial (Tables 4 and 6), 6 were decreased (*P* ≤ 0.049; prolactin, ALT, albumin, creatine kinase, LDH, cholesterol, RBC) in HE steers, whereas 3 (ALP, AST, glucose) tended (0.10 ≥ *P* > 0.05.) to be decreased and 1 (ammonia) increased (*P* ≤ 0.077). However, only prolactin, albumin, and cholesterol were consistently affected in each of the three periods. Decreased serum prolactin is the most consistently observed, and accepted, indicator of cattle suffering from fescue toxicosis (2, 8, 17, 18). The accompanying decreased serum cholesterol also is consistent with that found by others for cattle suffering from fescue toxicosis and is thought to be caused by elevated body temperatures (19–21). Although shown to be consistently decreased through all periods of the current trial, albumin response to the consumption of HE forages can vary. For example, a number of studies report lower levels of albumin in the serum of cattle consuming HE and suggest that this response could be due to the reduced dietary intake, decreased uptake by the gut of AA for the biosynthesis of albumin, damaged tissue, or increased catabolism of albumin (9, 22, 23). Alternatively, others report increased albumin levels in cattle grazing HE forages, potentially due to dehydration (18).

As just described, of the nine analytes and cell types measured that had across-period treatment effects, only three were consistently affected for each of the three periods. To more discretely determine when metabolic alterations were concomitant with grazing HE forages (rather than a total across-period treatment effect after a whole summer of grazing), serum enzymes, and blood constituents were measured and analyzed within the three time periods over the 85-day trial. This analysis was critical to identify potential treatment effects that may have been dependent on the length of grazing, thus potentially identifying metabolic adaptations to the length of HE forage consumption. Of the blood analytes and cell types evaluated (Tables 4 and 5), 9/19 analytes and 5/8 had period-specific changes, putatively indicating period-dependent shifts in metabolic capacity as detailed below.

### Period 1

Although the cohort groups was relatively small (*n* = 9–10), the large reduction in ADG of HE steers occurred only during the first period. In the latter two periods, the growth response of the HE steers paralleled the LE steers. Thus, the HE steers showed an adjustment in rate of BW gain to the alkaloid challenge but without a compensatory growth response. Concomitantly, there was a decrease in blood concentrations of triglycerides, albumin/globulin ratio, eosinophils, and lymphocytes but an increase in bilirubin. It was only during the first period that the concentrations of both serum cholesterol (27%) and triglycerides (25%) were depressed. The initial more than sixfold (14% of LE steers) depression of the classic endophytic fescue identifier, prolactin, in HE steers is consistent with reduced triglyceride synthesis in the presence of ergot peptides (24) and with the overall correlation found between serum triglyceride and prolactin concentrations (Table 8). This evidence of altered lipid metabolism has been associated with reduced forage intake of high endophyte fescue (25, 26). Although we did not measure these parameters, DiMarco et al. (25) found steers that had been fasted, and then refed, recovered their initially depressed cholesterol levels. With regard to correlating prolactin and triglyceride levels, cattle consuming high levels of endophyte forages have been shown to possess more saturated fatty acids in subcutaneous and perinephric fat than those consuming low levels of endophyte (27, 28). In the current study, a decreased in ADG and triglycerides was found in the first period, whereas ADG and triglyceride levels had recovered by the second and third period. By contrast, consumption of alkaloids is thought to be the cause of the loss of stimulation for insulin receptors that are involved in lipid metabolism (24), consistent with the trial-long depressed cholesterol levels of HE steers.

The reduced serum albumin and the albumin/globulin ratio in HE steers vs. LE steers would signify that albumin synthesis within the liver was impaired. For the HE steers, the 20% increased bilirubin also observed in period 1 signifies that excretory function may have been partially curtailed. The 6.3% increase in RBC concentration for HE steers indicates that the senescence of erythrocytes would not be the major contributor to the increased bilirubin (23). Moreover, increased RBC in HE steers is inconsistent with the understanding that prolactin increases erythropoiesis (29), as prolactin is decreased in HE steers. The unaltered value of total protein and γ*-g*lutamyltransferase would suggest that even though some protein synthesis and excretory function may have been abridged, liver function was not uniformly impaired. The unaltered ammonia, blood urea nitrogen, creatinine, blood urea nitrogen:creatinine ratio, and creatine kinase between treatment groups indicates that nitrogen metabolism, urea synthesis, renal glomerular filtration function, and skeletal muscles were not influenced during period 1.

Eosinophils (55.2%) and lymphocytes (17.6%) numbers were decreased in HE steers in period 1, suggesting that immune function may have been impaired (30–32). The reduced level of eosinophils in these young HE steers also has been found in mature beef steers grazing endophyte-infected tall fescue (18).

### Period 2

In contrast to the severe reduction in BW gains of HE steers found during period 1, the ADG by HE steers (0.26 kg/day) did not differ from that of LE steers (0.27 kg/day). Although moderate, this rate of gain suggests that the HE steers seemed to have adjusted to the physiological challenge of consuming forages that contained about 20 times more ergot alkaloids than LE steers. However, despite an equal rate of BW gain, prolactin, albumin, and cholesterol levels remained suppressed, and RBC increased in HE steers during period 2. By contrast, the albumin:globulin ratio and bilirubin levels no longer differed, indicating that hepatic albumin synthesis was still impaired, but possibly not to the overall extent as in period 1. Between treatment groups, similar values for bilirubin, total protein, and γ*-*glutamyltransferase would implied that hepatic excretory capacity was not affected during this period and that liver damage did not occur.

Some aspects of energy metabolism also appeared to have been altered in period 2 as compared to period 1. Although HE steers no longer possessed reduced levels of triglycerides relative to LE steers, they continued to have lower serum cholesterol, levels that were below the clinical reference range (9, 15). This suggests that the HE steers may be utilizing their cholesterol to supplement the altered energy metabolism. In addition, the reduced LDH of HE steers during period 2 could be indicative of a reduction in the transformation of pyruvate to lactate or a reduced “shuttling” of lactate between skeletal muscles and the liver. In rats, the sub-chronic exposure of rats to environmental toxins caused hepatotoxicity and a decrease in the serum levels of LDH (33). The increased duration of exposure to the high concentration of ergot alkaloids may have led to the reduced LDH as well for the HE steers.

Also occurring during period 2, increased (34%) plasma ammonia in HE vs. LE steers suggests that ammonia capturing and recycling mechanisms were impaired in the HE steers (34–36). However, because blood urea nitrogen did not differ, urea synthesis likely was not altered in HE vs. LE steers. Moreover, the unaffected creatinine, blood urea nitrogen:creatinine ratio, and creatine kinase between treatment groups suggests that renal glomerular filtration function and skeletal muscle turnover also were similar in HE and LE steers during period 2.

### Period 3

In the final period, ADG of the steers consuming the HE forage was the same as for LE steers. However, for both groups of steers, the ADG achieved was about four times greater than during period 2 (1.4 vs. 0.27 and 1.3 vs. 0.26, respectively). Despite these treatments, similar and good rates of gain for grazing steers, prolactin, albumin, and cholesterol levels remained suppressed, and ammonia and RBC levels remained higher in HE vs. LE steers. The prolonged high endophyte exposure allowed for further adaption by the HE steers. During period 3, the activities of creatinine kinase, AST, ALT, ALP, LDH, glucose concentration, and number of monocytes decreased.

Unlike periods 1 or 2, creatinine kinase in period 3 was depressed by 35% for steers grazing the HE pasture. The decreased level of creatine kinase was presumably the result of suppressed expression in striated muscle and possibly cardiac tissue or reduced tissue mass (9). Also, unique to period 3 was the serum reductions of clinical markers (ALP, ALT, and AST) for impaired metabolism. A reduction in serum ALP, a common indicator of high endophyte exposure (7, 37, 38), is related to decreased bone and intestinal isoenzymes activities (39). The decreased ALP could also be the result of reduced intakes and absorption of nutrients vital for ALP function (18). The reduced serum ALT presumably represents reduced cytosolic ALT in hepatocytes (40, 41), whereas reduced AST typically is interpreted as increased mitochondrial turnover in hepatocytes (41–43) and striated muscles (44). The lack of change in the AST:ALT ratio and decreased content of both enzymes is indicative of a relative constant rate of tissue turnover with either decreased concentrations of AST and ALT in tissue or diminished tissue mass (9), again indicating a “healthy” but metabolically altered liver in HE vs. LE steers.

During period 3, the concentration of RBC continued to be greater in HE vs. LE steers by 7.1%, whereas the monocytes were 44.7% lower in HE vs. LE steers. Monocytes are vital for immune response, wound healing, and tissue homeostasis (31, 45, 46). This decreased concentration of monocytes is similar to the report of Saker et al. (31) and could be due to the accumulated stress from the effects of grazing HE vs. LE pastures.

### Serum Prolactin was Poorly Correlated with Blood Analytes

Prolactin is reported to affect many physiological processes besides lactation (47). Therefore, the second objective of this study is to evaluate the potential relationships between serum prolactin and measured blood analytes. In general, little evidence was found to indicate that HE-decreased serum prolactin concentrations were associated with other assessed blood analytes. This lack of correlation was unexpected given that prolactin is a multifunctional hormone known to exert metabolic effects on many tissues due to the ubiquitous expression of prolactin receptors (48). In this regard, it is interesting to note that prolactin levels were lowest for both LE and HE steers in period 3, the period of greatest ADG for both steer groups. However, the correlation between triglycerides and prolactin levels in HE steers could prove to be very important as an indicator of altered energy metabolism (48, 49).

## Conclusion

In summary, growth, clinical, and biochemical parameters that changed in response to grazing HE vs. LE pastures can be divided into three groups: those that were continually altered (serum prolactin, albumin, cholesterol, and RBCs), those that were initially altered and then “recovered” (ADG, triglycerides, albumin/globulin ratio, bilirubin, lymphocytes, and eosinophils), and, subsequently, those that developed after long-term exposure (AST, ALT, ammonia, creatine kinase, LDH glucose, and monocytes). These findings suggest that the differences in blood analyte profiles may be indicative of whether and when grazing young steers are adjusting, metabolically, to consumption of HE pastures. However, mechanisms responsible for initial loss of BW gain of HE steers that occurred in period 1, and the subsequent parallel rates of gain to LE steers, but not compensatory growth, awaits discovery. Unexpectedly, with the exception of triglycerides and creatine, blood analytes were poorly correlated with serum prolactin levels.

## Author Contributions

JJ, JB, and JM were responsible for the design and acquisition of the data. All authors (JJ, ML, JB, and JM) were responsible for the analysis and interpretation of the data, drafting and/or critical revision of the intellectual content, and are accountable for the accuracy and integrity of this work.

## Conflict of Interest Statement

The authors declare that the research was conducted in the absence of any commercial or financial relationships that could be construed as a potential conflict of interest.

## Abbreviations

ADG, average daily gain; ALP, alkaline phosphatase: ALT, alanine transaminase; AST, aspartate transaminase; BW, body weight; HE, high toxic endophyte-infected tall fescue; LE, low toxic endophyte tall fescue-mixed grass; LDH, lactate dehydrogenase; RBC, red blood cell.
